# A Hospital for Soldiers Suffering from Shock

**Published:** 1914-12-05

**Authors:** 

**Affiliations:** Chairman of the London Hospital.


### A HOSPITAL FOR SOLDIERS SUFFERING FROM SHOCK.

### By VISCOUNT KNUTSFORD, Chairman of the London Hospital.

Will you allow me to reply to the points raised in your leading article of the week before last? I am so entirely in agreement with you that if the existing hospitals for nervous diseases could accommodate the cases for which this special hospital is required it would have been unnecessary and a waste of money to start yet another appeal and yet another hospital. I should go farther and call the doing so something a great deal worse. But it may readily be believed of me that I did not take upon myself the burden of collecting money and organising a hospital for soldiers suffering from nervous shock until I was assured by men, who knew, that the accommodation provided by the existing hospitals was not what was needed for a great number of these poor fellows.

To have any chance of recovery they need absolute quiet and isolation, and they must have separate rooms. Cubicle accommodation is useless.

The "National"—I suppose the best hospital of its kind in the world—has no private rooms, and two members of its staff are amongst those who signed my letter appealing for funds to start this hospital and testifying to the need of it.

The West End Hospital in Welbeck Street, excellent as it is in many ways, is impossible for us. I visited it with the hope that possibly we might be able to make use of it. But it has no private rooms, and is too noisy. One of the staff himself told me that the patients complained of the noise from the traffic in Marylebone Lane, which its wards face. And another member of its staff is a signatory to my letter.

The Maida Vale Hospital has only fourteen private rooms. The managers naturally would not be prepared to hand all these over to us, and, if they did, fourteen would not be sufficient. And, moreover, only the staff of that hospital would presumably be allowed to treat the patients.

We seek for our patients more accommodation, a wider influence, and more varied treatment.

It should satisfy most people, it certainly satisfied me, to have the assurance of the men who signed my letter—whose names I attach—that such isolation accommodation was absolutely essential, and that, with the exception of the Maida Vale Hospital, and there only to a very limited extent, it was not obtainable at any of the three existing hospitals in London for the treatment of nervous diseases. The following is the list of signatories to my original appeal:—

Dr. Stanley Bousfield, Dr. Milne Bramwell, Dr. Douglas Bryan, Dr. F. W. A. Bryden, Dr. A. G. Buchanan, Dr. J. Campbell McClure, Dr. James Collier, Dr. Maurice Craig, Dr. David Forsyth, Dr. Bernard Hart, Dr. T. B. Hyslop, Dr. E. T. Jensen, Dr. Constance Long, Professor W. McDougal, Dr. Crichton Miller, Dr. T. W. Mitchell, Dr. Heatly Spencer, Dr. Purves Stewart, Dr. Lloyd Tuckey, Dr. Aldran Turner, Dr. Hugh Wingfield, and Dr. Maurice Wright.

### The New Hospital's Accommodation.

We have got a small lay committee of three good business men and myself; we have got a first-rate house given to us in a very quiet position on Kensington Green, which will accommodate between thirty and forty patients in separate rooms and a full staff; furniture for twenty of the rooms has been given. Without issuing any appeal other than the letter in the Press we have about £6,000 in cash and other promises of money and personal service which justify us in opening this house, which we hope to do in a few weeks. Whether we shall be able to run a house in the country depends on the result of the appeal. I think we shall.

There is no spirit of competition or rivalry with any existing hospital. "Dog does not eat dog." We are just doing different work, that's all, and I am sure we can all work happily together. This hospital will end with the war. If it establishes the usefulness of strict isolation or of any special treatment for these nerve-shattered men its ephemeral existence will not have been in vain, and it will have done good work apart from the number of patients cured.

### Dr. Ash's Letters.

P.S.—For some reason unknown to me this move of mine is antagonistic to Dr. Ash, and in the *Observer* and in The Hospital he waxes eloquent against me. I have answered his *Observer* letter in last Sunday's number. What has he to say in The Hospital? Listen to his weighty comment on our (the signatories of my letter and my) statement that merely cubicled accommodation is not enough isolation for these patients:

"One would imagine that after the din and turmoil of the modern battlefield the nerve-wracked soldier would find even a bed in an ordinary hospital a welcome haven of rest. How much more so, then, would he feel the benefit of rest in a separate cubicle . . ."

Did anyone ever read such distilled rubbish? He might as well ask whether after the disturbance of being run over by a motor-'bus one would not find sufficient rest on a police ambulance without going to a hospital. "Rest" is not all that is needed in either case. Dr. Ash has not taken the trouble to ask what sort of cases are to be treated, and seems totally ignorant on this point. So it is a pity he should have tried to throw cold water on the scheme. But his letters have given me the opportunity of advertising it free.

Knutsford.

